# Natural Products: A Promising Therapeutics for Targeting Tumor Angiogenesis

**DOI:** 10.3389/fonc.2021.772915

**Published:** 2021-10-22

**Authors:** Ruyi Li, Xin Song, Yanan Guo, Peng Song, Dongzhu Duan, Zhe-Sheng Chen

**Affiliations:** ^1^ Department of Respiratory and Critical Care Medicine, The First Affiliated Hospital of Zhengzhou University, Zhengzhou, China; ^2^ School of Life Science and Engineering, Lanzhou University of Technology, Lanzhou, China; ^3^ Research Center of Traditional Chinese Medicine in Gansu Province, Gansu University of Chinese Medicine, Lanzhou, China; ^4^ Key Laboratory of Prevention and Treatment for Chronic Diseases by Traditional Chinese Medicine in Gansu Province, Affiliated Hospital of Gansu University of Chinese Medicine, Lanzhou, China; ^5^ Shaanxi Key Laboratory of Phytochemistry and College of Chemistry & Chemical Engineering, Baoji University of Arts and Sciences, Baoji, China; ^6^ College of Pharmacy and Health Sciences, St. John’s University, Queens, NY, United States

**Keywords:** tumor angiogenesis, natural products, angiogenic factors, endothelial cell apoptosis, anti-angiogenic mechanism, chemical structures

## Abstract

Tumor-associated angiogenesis is a key target for anti-cancer therapy. The imbalance between pro-angiogenic and anti-angiogenic signals elicited by tumor cells or tumor microenvironment always results in activating “angiogenic switch”. Tumor angiogenesis functions in multi-aspects of tumor biology, including endothelial cell apoptosis, tumor metastasis, and cancer stem cell proliferation. Numerous studies have indicated the important roles of inexpensive and less toxic natural products in targeting tumor angiogenesis-associated cytokines and apoptotic signaling pathways. Our current knowledge of tumor angiogenesis is based mainly on experiments performed on cells and animals, so we summarized the well-established models for angiogenesis both *in vitro* and *in vivo*. In this review, we classified and summarized the anti-angiogenic natural agents (Polyphenols, Polysaccharides, Alkaloids, Terpenoids, Saponins) in targeting various tumor types according to their chemical structures at present, and discussed the mechanistic principles of these natural products on regulating angiogenesis-associated cytokines and apoptotic signaling pathways. This review is to help understanding the recent progress of natural product research for drug development on anti-tumor angiogenesis.

## Highlights

1. The primary mechanism of tumor angiogenesis was depicted.
2. The current *in vivo* and *in vitro* models toward targeting angiogenesis were recapitulated.
3. Natural agents as therapeutics against tumor angiogenesis were summarized.

## Introduction

Angiogenesis refers to the process of new blood vessel formation from pre-existing endothelial cells in established vessels. The main cellular element of newly formed capillaries is endothelial cells (ECs), that are primarily modulated by vascular endothelial growth factor (VEGF) (1). After stimulating by VEGF, ECs can migrate to the lead of growing capillaries, that are referred to as tip cells. Upon the transformation of ECs to tip cells, the following ECs (stalk cells) will proliferate and maintain the structural and functional integrity of nascent vessels (2). Unlike physiological condition, tumor cells exposed to hypoxic condition secrete high levels of pro-angiogenic factors, such as VEGF. VEGF is the key regulator of the proper development of tumor blood vessels. Members of the angiopoietins, platelet-derived growth factor (PDGF-B), transforming growth factor (TGF-β) families and basic fibroblast growth factor (bFGF) are also significant factors in the formation of an immature vascular network in tumors (3). Moreover, recent literature has demonstrated that a variety of pro-angiogenic factors (VEGF, angiopoietin-1, bFGF) have the ability of inhibiting EC apoptosis (4) in tumor angiogenesis.

Natural products isolated from plants have been used as medicine from ancient times. The discovery of natural products for treatment dated back to 2600 BC in Mesopotamia (5). Traditional Chinese medicine (TCM) and Indian Ayurveda system are well documented over thousands of years. Early experiments have verified their properties of anti-inflammatory, anti-allergic and anti-infectious diseases (6, 7). Natural phytochemicals have been applied to the therapy of various diseases, including tumors (8). These phytochemicals can effectively inhibit the initiation, development and progression of neoplasm by regulating cellular proliferation, apoptosis and metastasis (9). Recent studies have indicated the important roles of natural compounds in modulating tumor angiogenesis by promoting the apoptosis of endothelial cell and inhibiting the angiogenesis-associated cytokines (10). In this review, we recapitulate the process of tumor angiogenesis and present some natural products that have the effect of anti-tumor angiogenesis.

A systematic literature search was conducted in PubMed database from 2010 to 2020. The following search terms were used: “natural products (or) natural product (or) polyphenols (or) polysaccharides (or) alkaloids (or) terpenoids (or) saponins” and “tumor angiogenesis (or) anti-angiogenic (or) angiogenic factors (or) vascular endothelial cells”. There were no language restrictions, and the abstracts of the papers identified by the initial search were evaluated by the lead reviewer Peng Song for appropriateness to this review. The inclusion criteria of references was that the abstracts of the references introduced the molecular mechanism investigation of natural products against tumor angiogenesis through *in vitro* or *in vivo* experiments.

## Physiological Vasculature

The function of an extensive vascular network is to supply nutrients and oxygen and remove metabolic waste. During early embryogenesis, vascular growth is the results of combination of vasculogenesis and angiogenesis (11). Vasculogenesis refers to that new blood vessels are formed from primitive ECs precursors, as opposed to angiogenesis, in which the blood vessels developed from pre-existing ECs in established vessels (12). In adulthood, new blood vessels are rarely formed except in several physiological or pathological processes such as female reproductive cycling and wound healing. The sprouting, migration and proliferation of ECs are regulated by various cytokines, among them VEGF is a pivotal one (13).

## Neoplasm Angiogenesis

### Angiogenic Switch

At the early stage of neoplasm events, tumors present a balance between cell apoptosis and cell proliferation (14). Tumor cells produce pro-angiogenic factors, such as VEGF, PDGF and anti-angiogenic factors, such as angiostatin and thrombospondin. The secretion of these factors is in balance and tumors maintain at the “dormancy state”. However, once the average volume of a tumor exceeds 1-2 mm^3^, insufficient supply of oxygen and nutrients to tumor tissues will occur. Under hypoxic and/or acidic conditions, this balance is disturbed, and the tumors switch from a non-vascular to a vascular state, which turns on “angiogenic switch”. At this state, hypoxia inducible factor 1 alpha (HIF1-α) is able to bind to hypoxia-regulators and induce the production of VEGF and other pro-angiogenic factors (15). Oncogene Ras and the mutated tumor suppressor gene TP53 also mediate this pro-angiogenic effect. The synthesis of anti-angiogenic factors are found decreased in these tumors (16). Even if only 1% of tumor cells turns on the “angiogenic switch”, tumor growth occurs. In turn, the growth of neoplasm tissues promotes the initiation of angiogenesis (17).

### Activated Factors and ECs Apoptosis in Tumor Angiogenesis

The most well-known receptors for tumor angiogenesis on the membrane of endothelial cells are tyrosine kinase receptors with their co-receptor, neuropilin. These receptors bind to VEGF or bFGF. The transcription of several pro-angiogenic factors can be initiated by the second messenger cascade of these receptors. High levels of VEGF can be found in most tumor types (1, 18). VEGF exerts the key role in the formation of new blood vessels and the proliferation of ECs. There are three tyrosine kinase receptors of VEGF, VEGFR-1 to -3. VEGFR-2 is the most prominent receptor in angiogenesis, which binds to VEGFA with strong tyrosine kinase activity (19). Moreover, latent forms of VEGF ligands are separated in the extracellular matrix, that are regulated by the release and activation of extracellular matrix-degrading proteases (e.g., MMP-9) (20). The up-regulation of other pro-angiogenic signals, such as FGF family, is involved in sustaining tumor angiogenesis (3, 21). Besides the VEGF signaling pathway, other signaling pathways and associated factors are involved in tumor angiogenesis. Angiopoietin 1 (ANGPT1) and ANGPT2 are important ligands from the ANGPT family, that bind to the receptor tyrosine kinase TIE2 on the membrane of ECs. Since ANGPT1 acts as an agonist of TIE2, its function is to promote vessel maturation. The activation of TIE2 mediated by ANGPT2 can induce vascular disruption and increase angiogenesis (22). Another important system that regulates tumor angiogenesis is the Notch-Notch-ligand system. Endothelial-specific ligand Delta-like 4 (DLL4) mediates the formation of the tip and stalk cells following VEGF stimulation (23).

**Figure 1:**
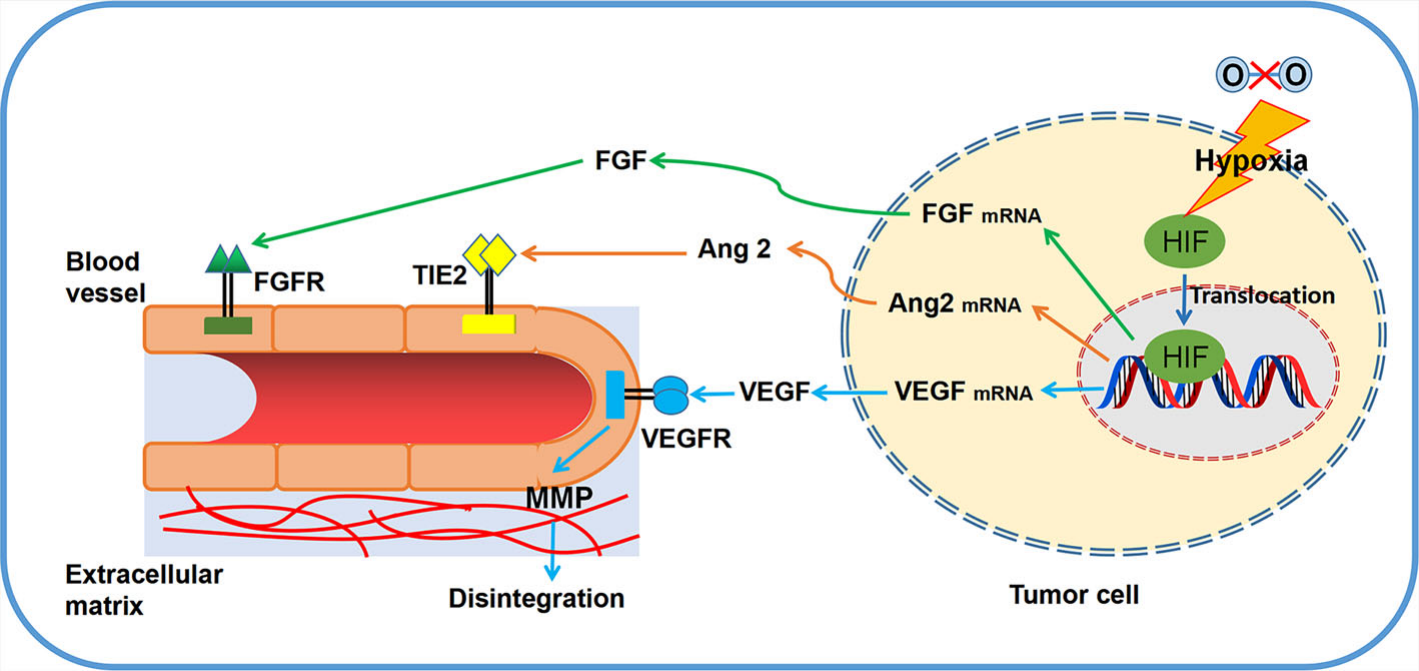
Primary principles of angiogenesis in tumor microenvironment. Several significant regulators (VEGF, FGF, MMPs, TIE2) and related pathways are involved in tumor angiogenesis.

**Figure 2:**
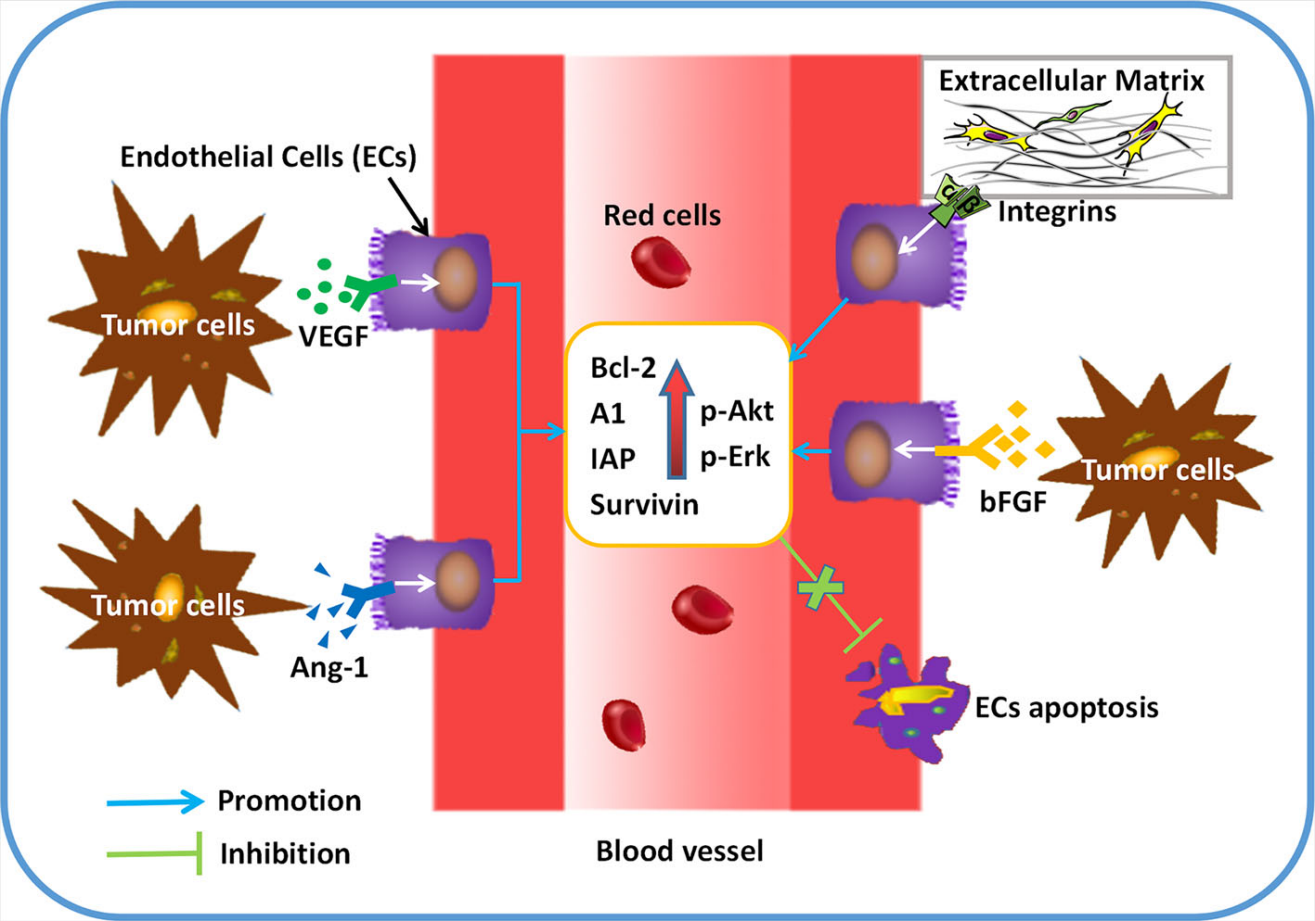
ECs apoptosis can be regulated by pro-angiogenic factors in tumor angiogenesis. After secreting a variety of cytokines (VEGF, bFGF, Angiopoietin-1), tumor cells exerts the role of anti-apoptosis in ECs by binding to its receptors. Up-regulated expression of anti-apoptotic proteins (BCL-2, A1, IAP, etc) and protein kinases (Akt, Erk, etc) can be found in ECs, which results in more survival and less apoptosis in ECs.

Apparently, neovasculature is strongly governed by ECs apoptosis. Both proangiogenic growth factors and extracellular matrix (ECM) act as the key roles in EC survival and angiogenesis (4). Induction of angiogenic growth factors and associated pathways are capable of inhibiting ECs apoptosis. For instance, ECs sustainment has been demonstrated to be linked with the modulation of cell apoptosis by VEGF (24). By increasing the expression of anti-apoptotic proteins, such as Bcl-2 and survivin, bFGF is able to decrease ECs apoptosis, which guarantees the cells survival (25). Of note, ANGPT1 involves in ECs apoptosis by modulating PI3K/Akt signaling and upregulating the anti-apoptotic protein surviving (26). Regarding the role of cell matrix and cell-cell interactions in angiogenesis, the anti-apoptotic effect of VEGF is dependent on the regulation of αvβ3-integrin and VE-cadherin protein (27, 28). Since the pivotal role of ECs in angiogenesis, many natural products are developed to target EC apoptosis. For example, resveratrol is able to increase Gly-LDL-induced vascular endothelial cell apoptosis (29). Similarly, *Physalis alkekengi L.* extract can reduce the apoptosis of ECs after exposing to hyperglycemia (30). Overall, angiogenic factors secreted by tumor cells (**Figure 1**) and EC apoptosis (**Figure 2**) are of significance in triggering and processing vessel formation in tumor microenvironment.

## Analysis of Vasculature in *In Vivo* and *In Vitro* Models

Vasculature has been explored for many decades and the application of *in vitro* and *in vivo* assays to evaluate the induction and inhibition of vascularization has exerted a significant role for researchers to study and understand angiogenesis (31). The well-established *in vitro* models, such as the model of ECs, are easily analyzed and reproducible, yet the cells become senescent in the culture after a few passages (32). Unlike the *in vitro* models, *in vivo* models, for example, the model of chick chorioallantoic membrane (CAM), owns the advantages of short experimental period and low cost, which is available for drug screening (33). As an ideal anti-tumor angiogenesis model, zebrafish has been widely used in drug screening due to its small size, transparent embryos, easy large-scale rearing, strong reproductive capacity and short experimental period (34). The aortic ring test is a model of angiogenesis based on organ culture. In this model, the molecular mechanism in regulating angiogenesis can be clarified, but it cannot truly reflect the microvascular environment of tumor growth (35). Another widely used *in vivo* model is xenograft tumor in mice. Of note, tumor angiogenic environment in animals is different from human, so assays in animals could not replace that in human (31). We have summarized the advantages and disadvantages of the current common angiogenesis models both *in vitro* and *in vivo*, which will provide clues for human cancer study (**Table 1**).

**Table 1:** Properties of common experimental models of angiogenesis *in vivo* and *in vitro*.

Assay type	Description	Advantages	Disadvantages	Refs
Endothelial cells	The proliferation of EC initializes the formation of blood vessel. Angiogenic-associated factors trigger the activation of EC, thereby degrading local basement membrane, promoting EC migration and invading the proximal ECM, which leads to EC sprout and capillary network formation. Therefore, vitro models of ECs were established to simulate angiogenesis in human body.	Simple, reproducible, and quantitatively analyzed.	Limited use for the senescent property of ECs after a few passages. Unrepresentative of the physiological reaction *in vivo*.	(32)
	Evaluates EC proliferation by MTT and cell cycle. Evaluates EC migration and invasion on a porous membrane with matrigel. Evaluates EC capillary network formation on the matrigel.			
Tumor cells	Tumor cells are able to secrete a variety of cytokines in tumor growth, which affect tumor angiogenesis. Therefore, we can indirectly evaluate the role of tumor angiogenesis by detecting the expression of related cytokines.	Specifically use for anti-angiogenic drugs development and drug detection of anti-angiogenic effects.	Unequal to human experiments because of the different local environment between tumor cell models and human models.	(36–38)
	Evaluates the expression of angiogenic factors secreted by tumor cells, such as VEGF, bFGF, etc. Evaluates the expression of matrix metalloproteinases secreted by tumor cells, such as MMP-2, MMP-9, etc.			
Chick chorioallantoic membrane (CAM)	CAMs is one of the most commonly used models to study the anti-angiogenic effect of drugs *in vivo*, which has been approved by FDA. At 6-8 days after hatching, the development of embryo membrane and vascular network is stable, but the immune system has not been completely established. Therefore, the embryo has no rejection to various foreign materials during this period, which makes it convenient to observe the effects of various drugs on angiogenesis.	Convenient to obtain materials and observe results. Short experimental period and low cost. Suitable for the screening of a large number of angiogenesis drugs.	The possibility of false positive results due to cellular damage, inflammatory reaction and fibrin degradation products	(33)
	Methods: The 8-day-old fertile eggs were opened from the end of the air chamber. After the opening, we can observe the growth of blood vessels on CAM and determine the best position of drug intervention, thereby avoiding the damage of chicken embryo caused by mechanical stimulation and eggshell detachment.			
Rat aortic rings	Aortic ring assay is a model of vascular regeneration based on organ culture. In this assay, the angiogenic vessels developed from a small segment of the aorta.	A wide range of materials and simple observation method Clear elucidation of angiogenic-associated molecular mechanism	Hardly reflection of the true microvascular environment of tumor growth	(35)
	Methods: the rat aorta was removed, cut into 1 mm vascular rings, embedded with fibrin glue or collagen glue, and then cultured in serum-free MCDB131 medium. During the cultural process, the number of new microvessels produced by aortic rings was calculated and analyzed quantitatively.			
Zebrafish	As an ideal anti-tumor angiogenesis model, zebrafish has been widely used in drug screening:	Small size, transparent embryos, easy large-scale rearing, strong reproductive capacity and short experimental period. Highly similar to the human genome sequence (87%)	____	(34)
	Zebrafish tumor is highly similar to human cancer at the levels of histology, gene expression and genome. This model can be used for imaging and high-throughput genetic screening, which provides a unique opportunity for cancer genome research *in vivo*. Using the techniques of forward genetics, reverse genetics, gene knockout, cancer cell transplantation and chemical inducers, we can conduct the replication of various zebrafish tumor models, the study of pathogenesis and the screening of antitumor drugs.			
Xenograft mice with tumor cells	The model of mice bearing with tumor cells is used to study the potential antitumor activity of drugs and their effects on tumor size (diameter, area or volume) and survival time of animals. This model can also be used to study the potential anti-angiogenic activity, uptake and distribution of drugs. If a drug has the anti-angiogenic effect, it will prevent or reduce the number of tumor neovascularization.	____	Irreplacement of the local environment of spontaneous tumor growth The differences about tumor growth environment between animal and human	(31)

## Natural Anti-Tumor Angiogenic Compounds Derived From Plants

### Polyphenols

**Figure 3:**
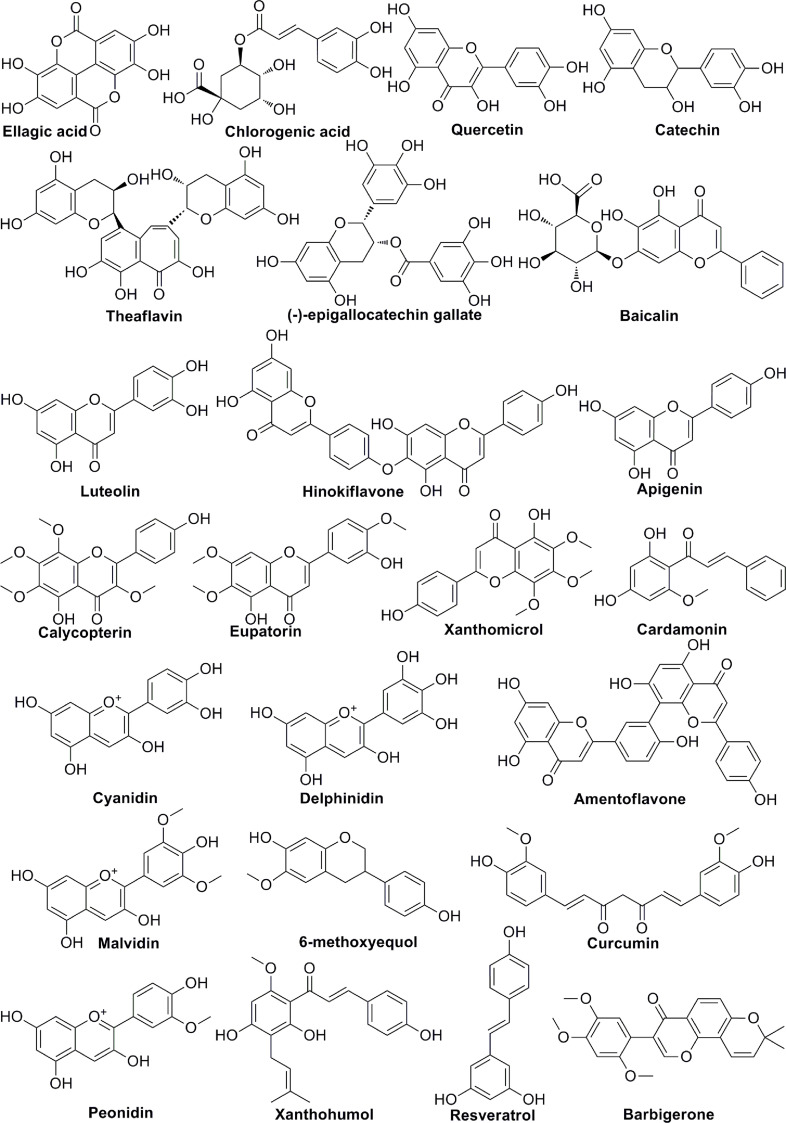
The chemical structures of polyphenols involved in tumor angiogenesis.

Polyphenols are members of a large family of chemical compounds with several phenolic groups, that can be found in foods and beverages of plant origin, including the spices, dried herbs, tea, red wine, coffee, cocoa products, seeds and nuts, vegetables, and fruits. Polyphenols are usually classified as phenolic acids, flavonoids, stilbenes, lignans, secoiridoids, among others. Polyphenols are of significance in the sustainment of human health. Recent studies have illustrated the pharmacologic functions of polyphenols that include antioxidation, vascular wall protection, improving enterogastric digestion, promoting immune response, and prevention of chronic disorders, such as tumor, atherosclerosis, hypertension, diabetes and so on. In addition to the summary of the chemical structures of polyphenols (**Figure 3**), the effect of several polyphenols (Ellagic acid, Chlorogenic acid, Quercetin, Catechin, Baicalin, Delphinidin, 6-methoxyequol, et al.) on tumor angiogenesis have also been studied in recent years (**Table 2**). Ellagic acid can inhibit angiogenesis by inhibiting the expression of VEGFR2 in ECV304 cell line. Moreover, researchers found that ellagic acid was able to regulate angiogenesis by inhibiting VEGF/VEGFR2, PI3K/Akt and MAPK signaling cascades in hamster cheek touch carcinogenesis model (39, 40). Chlorogenic acid is one of the hydroxygenic acid derivatives, which derives from *Eucommia ulmoides Oliv.* Chlorogenic acid suppressed tumor angiogenesis by blocking HIF-1α/Akt signaling pathway in lung cancer A549 cells (41). Quercetin, a flavanol-like compound, is derived from *Quercus iberica*. Quercetin reduces angiogenesis by inhibiting ERK phosphorylation and VEGFR-2 mRNA expression, and the calcineurin/NFAT signaling pathway (45, 46). Catechin is a flavanol-like compound and derived from black tea, which can affect angiogenesis by inhibiting VEGF (47). Baicalin, originated from *Scutellaria baicalensis Georgi*, can also inhibit tumor angiogenesis by regulating VEGFR (51). As for Delphinidin, it regulates angiogenesis by affecting the expression of HIF-1α and VEGF in A549 cells (56). 6-Methoxyquol is an isoflavone-like compound derived from soybean and can affect tumor angiogenesis through MAPK signaling pathway (59).

**Table 2:** Natural anti-tumor angiogenesis polyphenols and their sources, experimental models and anti-angiogenic mechanisms.

Category	Name	Source	Experimental model	Anti-angiogenic mechanism	Refs
Hydroxybenzoic acid derivative	Ellagic acid	blackberry	ECV304 cell line, Hamster cheek pouch carcinogenesis model, HUVEC cells	Inhibits VEGFR2 expression	(39, 40)
				Inhibits VEGF/VEGFR2, PI3K/Akt and MAPK signaling cascades	
Hydroxycinamic acid derivatives	Chlorogenic acid	*Eucommia ulmoides Oliv*	A549 cells	Suppresses HIF-1α/Akt pathway	(41)
	Curcumin	*Curcuma aromatica Salisb*	A549 and PC-9 cells, Nude mice xenograft tumor model, T24 and UMUC2 cells, HUVEC cells	Blocks c-Met expression and PI3K/Akt/mTOR pathway	(42–44)
				Blocks IGF2 and IGF2 mediated PI3K/AKT/mTOR Signaling Pathway	
				Cyclic nucleotide phosphodiesterases inhibition	
Flavonols	Quercetin	Quercus iberica	MCF-7 cells, BALB/c nude mice xenograft model, HUVEC cells, Transgenic zebrafish embryos TG	Inhibits calcineurin/NFAT pathway, Inhibits ERK phosphorylation and VEGFR-2 expression	(45, 46)
Flavanols	Catechin	black tea	HUVEC and HASMC cells	Inhibits VEGF expression	(47)
	Theaflavin	black tea	OVCAR-3 and A2780/CP70, HUVEC	TF1 reduces VEGF secretion in a HIF1α-independent manner, while the others in a HIF1α-dependent way	(48)
	(-)-epigallocatechin gallate	green tea	SW620, HT-29, HCT116, Endothelial cells and xenografts	Suppresses JAK/STAT3/IL-8 pathway and VEGFR2 expression	(49, 50)
Flavones	Baicalin	*Scutellaria baicalensis Georgi*	A549 cell, Xenograft tumors in nude mice	VEGFR expression inhibition	(51)
	Apigenin	*Apium graveolens L.var.dulce DC.*	PC3-M and LNCaP C4-2B cells	Blocks TGF-β1-Smad-VEGF pathway	(52)
	Luteolin	*Reseda odorata L*.	Sprague-Dawley rat	VEGF expression inhibition	(53)
	Eupatorin	Isatis tinctoria	HUVEC cells	VEGF expression inhibition	(54)
	xanthomicrol	*Citrus reticulata* Blanco	HUVEC cells and rat aortic rings	VEGF expression inhibition	(55)
	calycopterin	Dracocephalum kotschyi	HUVEC cells and rat aortic rings	VEGF expression inhibition	(55)
Anthocyanins	Delphinidin	*Pharbitis nil(L.)Choisy*	A549 cell	Inhibits HIF-1α and VEGF expression	(56)
	Cyanidin	purple sweet potato	HUVEC and HRMEC	Blocks ERK pathways	(57)
	Peonidin	lycium ruthenicum	HUVEC and HRMEC cells	Blocks ERK pathways	(57)
	Malvidin	*Malva sinensis Cavan.*	SCC131 cells	Suppresses JAK/STAT-3 pathway	(58)
Isoflavone	6-methoxyequol	soybean	HUVECs A431 cells, Mouse xenograft tumors model	Suppresses MAPK pathway	(59)
Pyranoisoflavone	Barbigerone	soybean	B16F10 melanoma cells, Zebrafish and mouse xenograft tumors models	Suppresses MEK 3/6/p38 MAPK signaling pathway	(60)
Chalcone	Cardamonin	Alpinia katsumadaiHayata	SKOV3 cells	Inhibis VEGF and HIF-α expression	(61)
	Xanthohumol	*Humulus lupulus L.*	BxPC-3 cells	Blocks NF-κB expression	(62)
Biflavonoid	Amentoflavone	Selaginella tamariscina	TSGH8301 cell	Suppresses VEGF expression	(63)
	Hinokiflavone	*Platycladus orientalis* (Linn.) Franco	CT26 cell	–	(64)
Stilbens	Resveratrol	Peanut	Rats	MMP-9 expression inhibition	(65)

### Polysaccharides

Polysaccharides are polymeric carbohydrate macromolecules, that are composed of long-chain monosaccharide units connected by glycosidic bonds. As pivotal bioactive macromolecules, polysaccharides are mainly extracted from plants, animals and microorganisms. Polysaccharides are currently applied to various biomedical products and functional foods because of their significant biological functions, such as inhibiting tumor growth, triggering immunity, antioxidation, anti-virus and neuroprotection. Recent studies indicated that polysaccharides extracted from higher plants showed anti-tumor activity by enhancing immunity. In addition to anti-tumor effect through immunomodulation, polysaccharides can directly inhibit tumor angiogenesis. Various polysaccharides, such as dandelion polysaccharide, sulfated polysaccharide, Fucoidan, LEP-2a, and Galactomannan, exert important roles in tumor angiogenesis (**Table 3**). Dandelion polysaccharide has been proved to inhibit angiogenesis both *in vivo* and *in vitro*. The PI3K/Akt signaling pathway is involved in this process, which inhibits the expression of VEGF and HIF-1α (66). Sulfurized polysaccharide is derived from *Phellinus ribis*. In the Lewis lung carcinoma (LLC) mouse model, sulfurized polysaccharide can inhibit vessel formation by modulating VEGF/VEGFR signaling pathway (67). Fucoidan, extracted from *Laminaria japonica*, regulates tumor angiogenesis by modulating MAPK and PI3K/Akt signaling pathways (68). Fucoidan is proved to reduce the expression of VEGF and PDGF in two chicken embryo chorioallantoic membrane (CAM) models (69). Moreover, in mice xenografted with DU-145 human prostate cancer cells, fucoidan can decrease tumor growth and angiogenesis by inhibiting JAK/STAT3/VEGF signaling pathway (70). Another important polysaccharide, Lep-2a, can restrain the expression of VEGF, CD105, bFGF, MMP-2 and MMP-9 in hepatocellular carcinoma (72). PSP001 is a galactomannan derived from the fruit rind of *Punica granatum L*. A recent study found that galactomannan was able to suppress the expression of VEGF, MMP-2 and MMP-9 and up-regulate the expression of TIMP-1 and TIMP-2 in cancer cells (74).

**Table 3:** Natural anti-tumor angiogenesis polysaccharides and their sources, experimental models and anti-angiogenic mechanisms.

Category	Name	Source	Experimental model	Anti-angiogenic mechanism	Refs
α-type polysaccharide	Dandelion Polysaccharide	the root of dandelion	HUVECs, CAM, Mice xenografted with Hepa1-6 and H22 cancer cells	Inhibits PI3K/AKT/HIF-1α/VEGF pathway	(66)
Sulfated polysaccharide	PRP-S16	*Phellinus ribis*	LLC (Lewis lung carcinoma) allografts tumor-bearing mice	Inhibits HIF-1α/VEGF/VEGFR-2/AKT pathway	(67)
A fucose-rich polysaccharide	Fucoidan	*Laminaria japonica, Fucus vesiculosus*	TNBC, HUVECs, CAM, Mice xenografted with DU-145 human prostate cancer	Suppresses MAPK and PI3K/AKT signaling pathway	(68–70)
				Inhibits VEGF and PDGF expression, Inhibits JAK/STAT3/VEGF pathway	
Homogeneous polysaccharide	HH1-1	safflower	CAM, BxPC-3 xenograft model and PDX model	Impedes the combination of EGFR and Galectin-3	(71)
				Inhibits Galectin-3/GFR/KT/OXO3 signaling pathway	
Exopolysaccharide	LEP-2a	*Lachnum* sp.	H22 (hepatocellular carcinoma) allografts tumor-bearing mice	Suppresses VEGF, CD105, bFGF, MMP-2 and MMP-9 expression	(72)
Water-soluble polysaccharide	PTP	the roots of *Polygala tenuifolia*	SKOV3 xenograft, Tumor growth in BALB/c mice	Decreases EGFR, VEGF, and CD34 expression	(73)
Galactomannan	PSP001	the fruit rind of *Punica granatum L.*	CAM, A375 and A549 cells, B16F10	suppresses the expression of VEGF, MMP-2 and MMP-9	(74)
				Upregulates the expression of TIMP-1 and TIMP-2	
Water-soluble polysaccharide	STPC2	*Sargassum thunbergii*	HUVECs, A549 cells	Downregulates MMP-2, VEGF and HIF-1α expression	(75)
Apigalacturonan-Rich Polysaccharide	ZCMP	The sea grass *Zostera caespitosa* Miki	HUVECs	–	(76)
Pectic polysaccharide	Corn pectic polysaccharide (COPP)	corn (*Zea mays* L.)	Mice were given B16F10 and injected through the lateral tail vein	Suppresses the expression of VEGF, MMP-2 and MMP-9	(77)
Arabinogalactan	Arabinogalactan	flowers of *Panax notoginseng*	BxPC-3, Pancreatic cancer cell xenograft tumor in nude mice, HMEC-1	Inhibits BMP2/Smad/Id1 signaling pathway	(78)
Huaier polysaccharide	TP-1	Huaier fungus	To establish an *in vivo* pulmonary metastasis model, SMMC-7721 cells were injected into BALB/c nude mice *via* the tail vein	Inhibits HIF-1α/VEGF pathway	(79)
	SP-1	*Trametes robiniophila* murr (Huaier)	Mice xenografted with SMMC-7721 human hepatocellular carcinoma cells	Suppresses the expression of VEGF, MMP-2, HIF-1α, STAT3 and MMP-9	(80)

### Alkaloids

**Figure 4:**
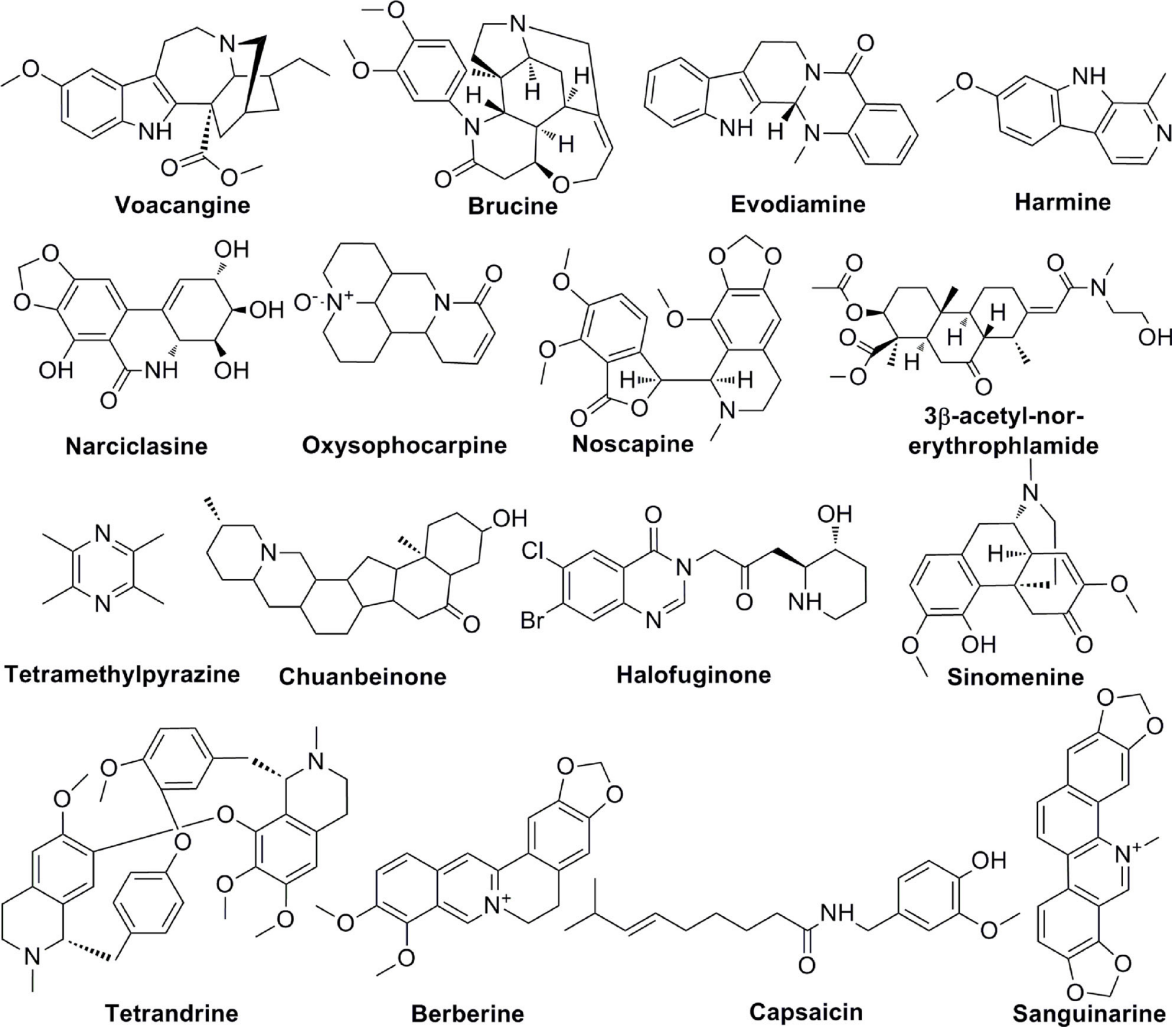
The chemical structures of alkaloids involved in tumor angiogenesis.

Alkaloids are a group of natural compounds with nitrogen structure and physiological function. Many alkaloids have complex nitrogen heterocyclic structures, and several compounds are organic amines with non-nitrogen heterocycles. Alkaloids have the properties of alkali, that are important active ingredients in Chinese herbal medicine. Although certain vitamins, amino acids and peptides from natural sources are also nitrogenous compounds, they are not included in the category of alkaloids. Alkaloids are ubiquitous in nature, especially in the plant kingdom. Alkaloids have been reported in at least 140 species of plants, especially in Papaveraceae, tetrandridae, Solanaceae, Leguminosae, Apocynaceae, Ranunculaceae, and tillering family, et al. The common alkaloids include pyrrole, pyridine, quinoline, isoquinoline, indole, imidazole, purine, anisodamine, terpenoids, steroids, organic amines, and so on and the chemical structures of these alkaloids are shown in **Figure 4**. Current studies proved the biological activities of alkaloids, including anti-tumor, anti-bacterial, anti-inflammatory, antiviral, antiarrhythmic, analgesic, and antispasmodic effects. Voacangine is an indole-like compound, which is rich in *Ervatamia Stapf*. Treatment with voacangine decreases the VEGFR2 kinase activity in athymic nude mice bearing glioblastoma tumors and HUVECs, suggesting that its anti-angiogenic role is closely associated with VEGFR2 (81). Evodiamine is an indoloquinazoline-like compound, which is derived from *Euodia rutaecarpa (Juss.)*. Evodiamine is able to modulate angiogenesis by inhibiting the expression of VEGF through the c-Mer/Src/STAT3 signaling pathway (82). By inhibiting β-catenin, which reduces VEGF-A expression, Le Shi et al. found the anti-angiogenic role of evodiamine (83). As an isocarbostyril compound, narciclasine regulates HUVECs proliferation by a RhoA-independent activation of the Rho kinase Rock (84). Tetromethylpyrazine is an amide alkaloid, which is extracted from *Ligusticum chuanxiong hort*. Tetromethylpyrazine combined with paclitaxel inhibited angiogenesis both *in vivo* and *in vitro* through blocking ERK1/2 and AKT signaling pathways (85). Previous studies found that harmine, a β-carboline alkaloid, suppressed bladder cancer growth by its anti-angiogenic effect, which was linked to VEGFR2 signaling pathway (86). Oxysophocarpine is a quinolizidine alkaloid and it reduces the levels of HO-1, VEGF, MMP-9 and HIF-1 in OSCC cells (87). 3-acetyl-norerthrophlamide is a cassaditerpene alkaloid, which comes from *Erythrophleum fordii*. 3-acetyl-norerthrophlamide and exerts the anti-angiogenic function by inhibiting the eNOS activation and NO production (88). Many alkaloids, such as chuanbeinone, halofuginone, tetrandrine, also play significant roles in tumor angiogenesis (**Table 4**).

**Table 4:** Natural anti-tumor angiogenesis alkaloids and their sources, experimental models and anti-angiogenic mechanisms.

Category	Name	Source	Experimental model	Anti-angiogenic mechanism	Refs
Indole	Voacangine	*Ervatamia Stapf*	Athymic nude mice bearing glioblastoma tumors consisting of U87MG glioblastoma, HUVECs	Inhibits VEGFR2 kinase activity and its downstream signaling by binding to the kinase domain of VEGFR2	(81)
	Brucine	*Strychnos nux-vomica Linn*	Human breast cancer cell line MDA-MB-231	Inhibits VEGF/VE-cadherin/EphA2/MMP-9/MMP-2 pathway	(89)
Indoloquinazoline alkaloid	Evodiamine	*Euodia rutaecarpa* (Juss.) Benth. (Rutaceae)	Human prostate cancer (PC-3 and DU145), Mouse xenograft model with HCC cell lines (HepG2, SMMC-7721, H22), HUVECs, The number of capillary sprouts from Matrigel embedded rat thoracic aortic rings	Inhibits c-Mer/Src/STAT3 pathway Inhibits VEGF and MMP-9 expression inhibits β-catenin expression	(82, 83)
Isocarbostyril alkaloid	Narciclasine	*Narcissus* and *Haemanthus* species	HUVECs	By the RhoA-independent activation of the Rho kinase ROCK downregulation of VEGFR2 expression	(84)
Amide alkeloid	Tetramethylpyrazine	*Ligusticum chuanxiong* hort	Ovarian cancer A2780 xenograft mouse models, HUVECs, SKOV3 cell	Inhibits ERK1/2 and Akt pathways	(85)
β-carboline alkaloid	Harmine	Pergamum harmala seeds	The xenograft mouse model assay of human bladder cancer used RT4 cell line, HUVECs, Rat aortic ring assay	Inhibits VEGFR2, ERK1/2 and Akt signaling pathways	(86)
Quinolizidine alkaloid	Oxysophocarpine	*Sophora flavescens* Ait. (Kushen), *S. alopecuroides L*. (Kudouzi or Kugancao), and other leguminous plants of the genus Robinia	HUVECs, BALB/c nude mice were injected with SCC-9 cells	Reduces the levels of HO-1, VEGF, MMP-9 and HIF-1α expression	(87)
Opium alkaloid	Noscapine	*Papaver somniferum L*	HUVECs	Inhibits growth of human endothelial cells, cord formation of human endothelial cells and chemotaxis factors responsible for the angiogenesis	(90)
Cassaine diterpene alkaloid	3β-acetyl-nor erythrophlamide	*Erythrophleum fordii*	HUVECs, A549 xenograft mouse model	Inhibits the VEGF-mediated eNOS activation and NO production	(88)
Isosteroidal Alkaloid	Chuanbeinone	Bulbus of *Fritillaria pallidiflora*	LLC and S180-bearing mice	Inhibits the expression of VEGF	(91)
quinazolinone alkaloid	Halofuginone	*Dichroa febrifuga* Lour.	NB4 and HUVEC cells, NOD/SCID mice were transplanted with leukemic cells	Reduces VEGF secretion and phosphorylation of SMAD-2	(92)
				Blocks TGF-β signaling pathway	
Bisbenzylisoquinoline alkaloid	Tetrandrine	the roots of the medicinal plant *Stephaniae tetrandrae S. Moore* (Han-Fang-Ji in Chinese)	Mouse endothelial cells (EOMA cell), Huh7 tumor xenografts	Inhibits EOMA cells proliferation *via* ROS/Akt pathway	(93)
Isoquinoline alkaloids	Berberine	*Coptis Rhizome*	MHCC-97L cells xenograft mouse model	Inhibits the expression of Id-1, VEGF	(94)
	Sinomenine	*Sinomenium actum Rehd.et wils.*	HUVEC, HOS-Luc cells xenograft mouse model	Inhibits the expression of VEGF, MMP-2, MMP-9, RANKL	(95)
				Inhibited invasion and metastasis *via* suppressing the CXCR4-STAT3 pathway	
Benzophenanthridine alkaloid	Sanguinarine	the root of *Sanguinaria canadensis* and other poppy-fumaria species	Human microvascular endothelial cells (HMVECs), A549 cells	Inhibits the activation of serum starvation and hypoxia-induced VEGF promoter activity	(96)
Amide alkaloids	Capsaicin	*Capsicum annuum L.*	HUVEC, A549	Re-activation of p53-SMAR1, inhibits the expression of HIF-1α and Cox-2, down regulate VEGF	(97)

### Terpenoids

**Figure 5:**
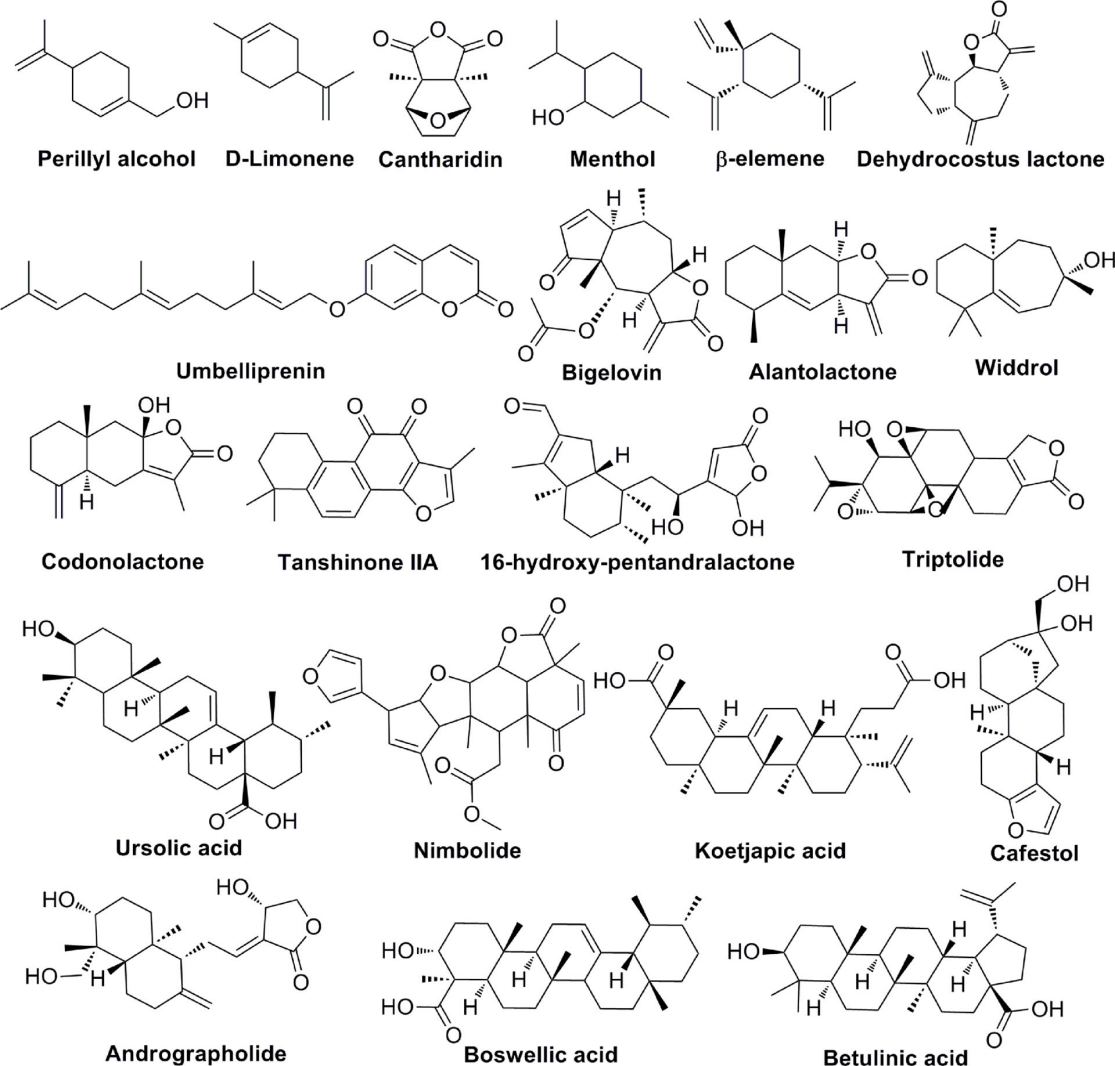
The chemical structures of terpenoids involved in tumor angiogenesis.

Terpenoids are natural products that derive from mevalonic acid. Terpenoids are composed of multiple isoprene (C5) units with the general formula of (C_5_H_8_)_n_. More than 20,000 terpenoids exist in nature and are widely distributed in the plant kingdom, such as Compositae, Ranunculaceae, Araliaceae, Oleaceae, Magnoliaceae, Lauraceae, Aristolochiaceae, and more. Based on the different molecular structures (**Figure 5**), terpenoids are divided into five groups: monoterpenes, sesquiterpenes, diterpenoids, triterpenoids and terpenes compound. Most of the bioactive terpenoids have been isolated from medicinal plants. For example, monoterpenes and sesquiterpenes are extracted from essential oils of medicinal plants and triterpenes are primarily available in balsams and resins. Recent studies have found that terpenoids have a variety of biological functions, including the regulation of enzyme system, cell surface signaling transduction, immune function, cell differentiation, tumor proliferation and angiogenesis. Important terpenoids (Perillyl Alcohol, β-elemene, Alantolactone, Tanshinone IIA, Triptolide, Ursolic acid, Koetjapic acid, et al) and their molecular signaling pathways participate in modulating tumor angiogenesis (**Table 5**). Perillyl Alcohol, hugely available in the essential oils of several plants (Lavendin, Mints, Cherries, etc.), possesses the anti-angiogenic property, the mechanism of which is probably to decrease the release of VEGF in cancer cells and stimulate the expression of Ang2 in endothelial cells (98). β-elemene is a sesquiterpene compound isolated from *Curcuma Zedoaria*. It inhibits the proliferation and metastasis of melanoma by suppressing VEGF-mediated angiogenesis (102). Alantolactone is also a sesquiterpene compound extracted from *Inula Helenium.* Alantolactone inhibits angiogenesis by reducing the phosphorylation of VEGFR2 and its downstream protein kinases, including PLCγ1, FAK, Src, and Akt in breast cancer (106). Tanshinone IIA is the key compound of Diterpene, isolated from *Salvia Miltiorrhiza Bunge*. Tanshinone IIA has anti-angiogenic activity in human EPCs, which is associated with the regulation of VEGF, PLC, Akt and JNK signaling pathways (109). Triptolide is also a Diterpene compound, derived from *Tripterygium Wilfordii*. Triptolide exerts the anti-angiogenic role in breast cancer by inhibiting ERK1/2-HIF1-α-VEGFA signaling pathway both *in vitro* and *in vivo* (113). As an important Triterpene compound, ursolic acid is able to decrease the expression of VEGF and iNOS in ehrlich ascites carcinoma (114). Koetjapic acid is also a significant Triterpene compound. Koetjapic acid is found to reduce the expression of VEGF in various angiogenic models, such as rat aortic ring, CAM and HUVECs (116).

**Table 5:** Natural anti-tumor angiogenesis terpenoids and their sources, experimental models and anti-angiogenic mechanisms.

Category	Name	Source	Experimental model	Anti-angiogenic mechanism	Refs
Monoterpene	Perillyl alcohol	the essential oils of several plants (lavendin, mints, cherries, etc.)	CAM, HUVECs, BLMVECs, K562	Inhibits VEGF and Akt pathways	(98)
	D-limonene rich volatile oil	blood orange (*Citrus sinensis* (L) Osbeck)	SW480 and HT-29 cells, HUVECs	Reduces the levels of VEGF, MMP-9 expression, inhibits the activation of VEGFR1	(99)
	Cantharidin	Mylabris	Subcutaneous and orthotopic pancreatic xenograft models with PANC-1 cells	Inhibits ERK, JNK, PKC, and NF-κB pathways	(100)
	Menthol	*Mentha haplocalyx Briq*	HepG2 cells	Inhibits the expression of VEGF	(101)
Sesquiterpene	β-elemene	the essential oil of *Curcuma zedoaria*	Rat aortic ring, Chick embryo chorioallantoic membrane, Melanoma growth and metastasis assay in C57BL/6 mice	Suppresses VEGF pathway	(102)
	Dehydrocostus lactone	Saussurea lappa and Laurus nobilis	lung cancer cells (A549 and H460 cells), Matrigel plugs were implanted in C57BL/6 mice	Suppresses of HIF-1a, Akt and pAkt, GSK-3β and pGSK-3β, as well as ERK, pERK, mTOR, and p-mTOR	(103)
				Inhibits MMP-2 and MMP-9	
	Umbelliprenin	Different spices of *Ferula* of umbeliferace	CT26 tumor cells, L929 cells	Inhibits the expression of VEGF, MMP2, MMP-9 and CD31	(104)
				Potentiates immune response by IFN-γ increment and IL-4 decrease	
	Bigelovin	*Inula helianthus-aquatica*	HCT 116 cells, Orthotopic tumor allografts and experimental metastatic models with colon 26-M01 cells	Interferes IL6/STAT3 and cofilin pathways	(105)
	Alantolactone	*Inula helenium*	HUVECs, human MDA-MB‐231 breast cancer xenograft in mice	Suppresses the phosphorylation of VEGFR2 and its downstream protein kinase including PLCγ1, FAK, Src, and Akt	(106)
	Codonolactone	*Atractylodes lancea*	HUVECs, EA.hy 926 cells	Inhibits MMPs expression and VEGF secretion by down-regulating BMP/Runx2 activation	(107)
	Widdrol	*Juniperus chinensis*	HUVECs, Human colon adenocarcinoma HT29 cells	Suppresses phosphorylation of VEGFR2, AKT, FAK and eNOS	(108)
Diterpene	Tanshinone IIA	The dried root of *Salvia miltiorrhiza* Bunge	Human EPCs, CAM, Matrigel plug model	Inhibits EPC angiogenesis through the VEGF, PLC, Akt and JNK signaling pathways	(109)
	16-hydroxy-pentandralactone	*Vitex cofassus*	HUVECs	Inhibits VEGF-stimulated HUVEC proliferation	(110)
	Cafestol	Unfiltered coffee	HUVECs	Inhibits the phosphorylation of FAK and Akt and decreases nitric oxide production	(111)
	Andrographolide	*Andrographis paniculate*	HUVECs, A549 Cells	Inhibits MMP-9 expression	(112)
	Triptolide	*Tripterygium wilfordii*	HUVECs, Breast cancer cells (Hs578T and MDAMB231)	Inhibits the ERK1/2-HIF1-α-VEGFA signaling pathway	(113)
Triterpene	Ursolic acid	Many plant foods	Ehrlich ascites carcinoma (EAC) cells, Swiss albino mice with EAC cells	Inhibits VEGF and iNOS expression	(114)
	Nimbolide	*Azadirachta indica* leaves	HCT-116, HT-29, Caco-2, CRC cells	Inhibits VEGF and MMP-9 expression	(115)
	Koetjapic acid	*Sandoricum koetjaoe* Merr	rat aortic ring, CAM, HUVECs	Inhibits VEGF expression	(116)
	Boswellic acid	*Boswellia serrata*	Ehrlich ascites carcinoma (EAC) cells, Swiss albino mice with EAC cells	Inhibits VEGF and TNF-α expression	(117)
	Betulinic acid	The stembark of *Betula* ssp. and from many other plants	Human endometrial adenocarcinoma (EA) cells	Inhibits prolidase, HIF-1α and VEGF expressions	(118)

### Saponins

**Figure 6:**
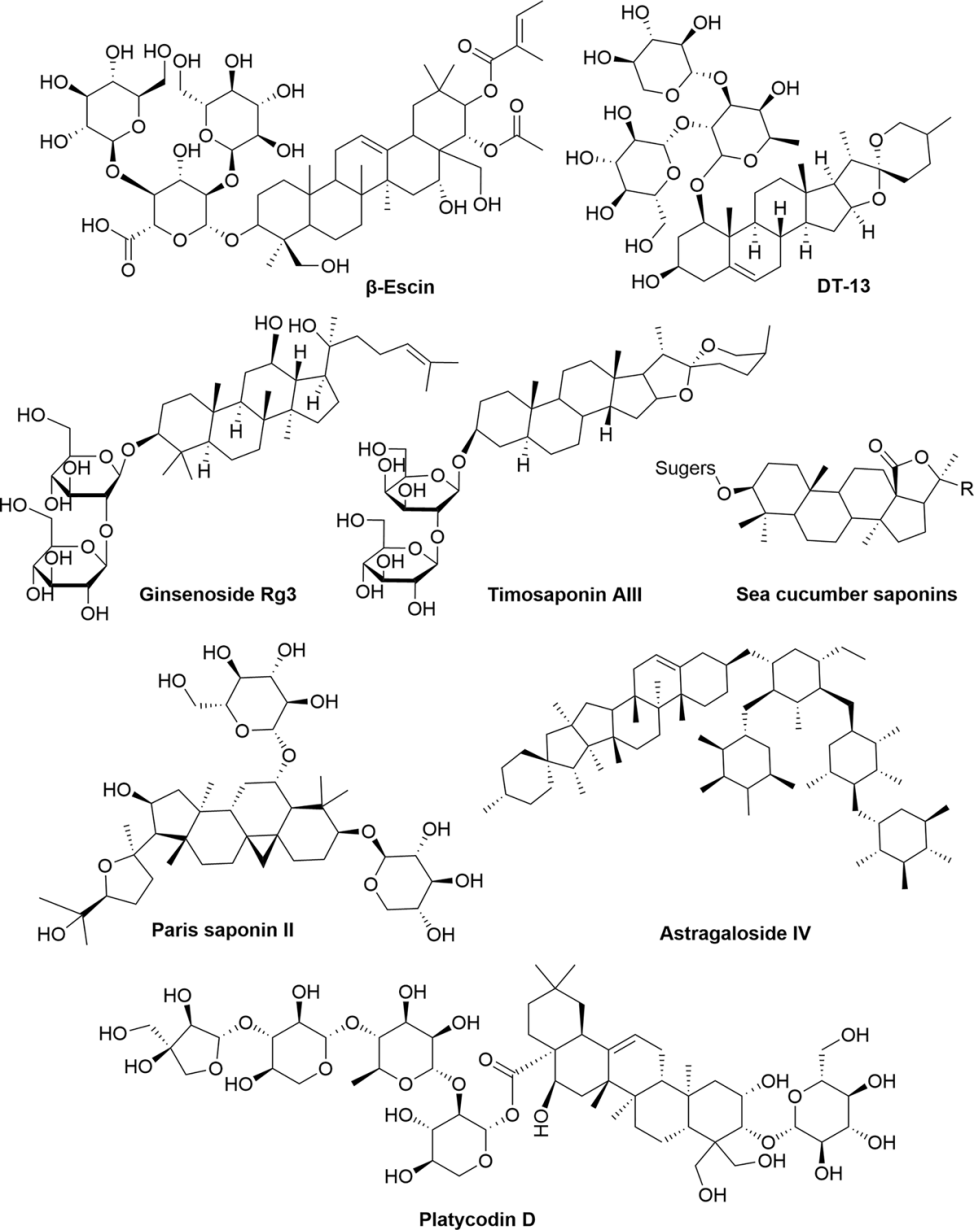
The chemical structures of saponins involved in tumor angiogenesis.

Saponins are composed of sapogenins and sugars. The sapogenins are triterpenes or spirostanes and the make-up sugars are commonly glucose, galactose, rhamnose, arabinose, xylose, glucuronic acid and galacturonic acid. Lipophilic sapogenins and hydrophilic sugar chains make the saponin an excellent surfactant. Saponins are mainly distributed in terrestrial higher plants and marine organisms, such as starfish and sea cucumber. Many Chinese herbal medicines, such as *Ginseng, Polygala Tenuifolia, Platycodon grandiflorum, licorice, Anemarrhena and Bupleurum*, contain saponins as the main active ingredients. According to the different structures of sapogenins, saponins have the biological functions of anti-tumor, hypoglycemic, cholesterol lowering, liver protection, immune regulation, anti-inflammatory and anti-microbial effects. The chemical structures (**Figure 6**) and anti-angiogenic property of several saponins (Timosaponin AIII, Ginsenoside Rg3, β-Escin, sea cucumber saponins, et al) has been identified in recent years (**Table 6**). Timosaponin AIII is a steroidal saponin, derived from *Anemarrhena asphodeloides Bge*. It exerts anti-angiogenic effect through VEGF/PI3K/Akt/MAPK signaling pathway in the zebrafish embryos model (119). Ginsenoside Rg3 is a triterpene saponin, isolated from *Panax ginseng*. Ginsenoside Rg3 can decrease the expression of MMP-2, MMP-9 and VEGF in B16 cell tumor mouse model and *in vitro* B16 cell model (122). β-escin is also a kind of triterpene saponin, extracted from *Aesculus hippocastanum* seeds. β-escin can inhibit melanoma angiogenesis by increasing the expression of TIMP-1 and TIMP-2 (123). Sea cucumber saponins are crude saponins, that are prevalent in Holothuria leucospilota. The saponins extracted from sea cucumber can inhibit the expression of VEGF-D and TGF-β in MCF7 cells (126).

**Table 6:** Natural anti-tumor angiogenesis saponins and their sources, experimental models and anti-angiogenic mechanisms.

Category	Name	Source	Experimental model	Anti-angiogenic mechanism	Refs
Steroidal saponin	Timosaponin AIII	*Anemarrhena asphodeloides* Bge	Zebrafish embryos, HUVECs	Inhibits VEGF/PI3K/Akt/MAPK signaling cascade	(119)
	DT-13	Dwarf lilyturf tuber	HUVECs, CAM	Inhibits the levels of p-VEGFR-2, p-ERK1/2 and p-Akt, Inhibits the expression of VEGF	(120)
	Paris saponin II	*Rhizoma paridis*	HUVECs, SKOV3 cells	Suppresses NF-κB signaling	(121)
Triterpene saponin	Ginsenoside Rg3	*Panax ginseng*	B16 melanoma cells, C57BL/6 mice with B16 cells	Inhibits the expression of MMP-2, MMP-9 and VEGF	(122)
	*β*-Escin	*Aesculus hippocastanum* seeds	B16F10 and SK-MEL5 cells	Increases the expression of TIMP-1 and TIMP-2	(123)
	Astragaloside IV	*Astragali radix*	C57BL/6 mice with LLC cells	Blocks the M2 polarization of macrophages partially through the AMPK signaling pathway	(124)
	Platycodin D (PD)	the roots of Platycodon grandiflorum	C57BL/6 mice with H22 cells	Inhibits the expression of VEGF	(125)
Crude saponins	Sea cucumber saponins (SCS)	*Holothuria leucospilota* (sea cucumber)	MCF7 cells	Inhibits the expression of VEGF-D and TGF-β	(126)
	Rh.Sp saponins	*Rumex hastatus* D. Don.	CAM	–	(127)

## Conclusions

This review article shed a light on the role of natural compounds in cancer therapy by modulating angiogenic factors and ECs apoptosis. In addition, the chemical structures of natural compounds involved in tumor angiogenesis were summarized and shown in supplementary material with the format of CS ChemDraw Drawing, which could be conveniently used for the related researchers. Natural products have presented as new stars in the field of anti-tumor neovascularization research for their easy availability and cost-effectiveness (128). A large number of epidemiological studies proved the reduction of cancer incidence upon the high nutritional ingestion of vegetables and fruits (129). Moreover, natural products emerged as SIRT6 modulators can be successfully applied to treat cancer, inflammation, Alzheimer’s Disease, etc (130).

Currently, anti-angiogenic drugs are widely used and recognized in cancer treatments because they enriched the arsenal of chemotherapeutic drugs. The majority of modern targeted drugs fail to achieve the expected therapeutic effects. Consequently, the anti-cancer regimens have shifted to multi-targeted therapies using traditional and integrative natural products. There is a huge number of natural products for anti-angiogenic substances and the study on the complex mechanisms of these compounds is just started. Studies on the role of different structures of natural compounds in inhibiting tumor angiogenesis would assist in anti-cancer drug discovery and development. The anti-angiogenic therapy of human cancer patients is based on pre-clinical models that simulate the pathogenesis of human cancers, and most of them are elucidated in this review. It is certain that future patients will be benefited from the novel discoveries of natural products, thus a lot of research works are warranted.

## Perspectives and limitations

As for clinical application of natural products in tumor, issues have emerged during the past few decades. The bioavailability of natural products is the major restriction, since these compounds have the properties of poor aqueous solubility and low absorption rate. Prior to the use of natural products in anti-tumor therapy, the concentration problem needs to be resolved. At present, several carriers (nanoparticles, micelles, lipids, etc) with natural products are being developed to apply to the delivery of these compounds to human body systems. Therefore, as hopeful therapeutic targets towards tumor angiogenesis, more efforts should be made to the development of natural compounds and their modifiers according to their molecular mechanisms and involved signaling pathways in tumors.

## Author Contributions

PS, Z-SC, and DD conceived and designed the review. RL and XS created the tables and figures, and wrote the draft manuscript. RL, XS, and YG revised the manuscript and performed the table design. PS, Z-SC, and DD reviewed and edited the manuscript. All authors read and approved the final manuscript.

## Funding

This work was supported by the National Natural Science Foundation of China (81803779); the Gansu Province Science Foundation for Distinguished Young Scholars (20JR10RA348) and the Gansu Province Science Foundation for B Program (1508RJZA018) from Gansu Provincial Sci. & Tech. Department; the Open Project of Research Center of Traditional Chinese Medicine, Gansu Province (zyzx-2020-zx1); the Open Project of Key Laboratory of Prevention and Treatment for Chronic Diseases by TCM in Gansu Province (GSMBKY2015-01); the Gansu Province Health Industry Scientific Research Program Management Project (GWGL2014-53); and the National Natural Science Foundation of Shaanxi Province (2018JQ2051).

## Conflict of Interest

The authors declare that the research was conducted in the absence of any commercial or financial relationships that could be construed as a potential conflict of interest.

## Publisher’s Note

All claims expressed in this article are solely those of the authors and do not necessarily represent those of their affiliated organizations, or those of the publisher, the editors and the reviewers. Any product that may be evaluated in this article, or claim that may be made by its manufacturer, is not guaranteed or endorsed by the publisher.
